# Diffuse hemorrhagic septic cerebral emboli complicating infective endocarditis: A radiology case report

**DOI:** 10.1016/j.radcr.2026.04.094

**Published:** 2026-05-23

**Authors:** Hind Qajia, Saleck Choumad, Maryam Lejouad, Yassine Eddahoumi, Ittimade Nassar, Kaoutar Imrani

**Affiliations:** a Central Radiology Department, Ibn Sina University Hospital, Rabat, Morocco; b Faculty of Medicine and Pharmacy of Rabat, Mohammed V University, Rabat, Morocco

**Keywords:** Infective endocarditis, Septic emboli, Brain MRI, Intracranial hemorrhage, SWI

## Abstract

Neurological complications are frequent in infective endocarditis and significantly impact prognosis. Septic cerebral emboli may result in ischemic or hemorrhagic lesions, often multifocal. We report the case of a 40-year-old patient admitted to the intensive care unit for infective endocarditis who developed impaired consciousness. Brain magnetic resonance imaging (MRI) demonstrated multiple supratentorial and infratentorial hemorrhagic lesions consistent with diffuse septic emboli, associated with subarachnoid hemorrhage and a small subdural hematoma. This case highlights the key role of multiparametric MRI in the diagnosis and assessment of neurological complications of infective endocarditis.

## Introduction

Infective endocarditis (IE) remains a severe disease with high morbidity and mortality despite advances in medical and surgical management. Neurological complications occur in up to 40% of patients and are among the most serious extracardiac manifestations [1,2]. They mainly result from septic embolization to the cerebral circulation, leading to ischemic infarcts, hemorrhagic transformation, intracranial hemorrhage, or infectious aneurysms [3].

Brain MRI is the imaging modality of choice for detecting and characterizing these lesions. We report a case of diffuse hemorrhagic septic cerebral emboli demonstrated on MRI in a patient with infective endocarditis.

## Case report

A 40-year-old patient was admitted to the intensive care unit for documented infective endocarditis. During hospitalization, the patient developed an acute impairment of consciousness, prompting urgent brain MRI.

Brain MRI revealed:

• Multiple diffuse punctate and lacunar lesions involving both supratentorial and infratentorial compartments, hyperintense on T2 and FLAIR sequences, with diffusion restriction, and hypointense on SWI, indicating hemorrhagic components.
• A right frontal lesion measuring 29 × 19 mm associated with sulcal hemorrhagic suffusion and a thin right fronto-parietal subdural hematoma with a maximal thickness of 3 mm, responsible for mild mass effect and a leftward midline shift of approximately 4 mm. This may also be explained by sepsis-related coagulopathy despite normal initial coagulation tests.
• A left cerebellar lesion measuring 19 × 15 mm with minimal hemorrhagic sulcal involvement.
• Global midline shift estimated at 3 mm.

**Fig. 1:**
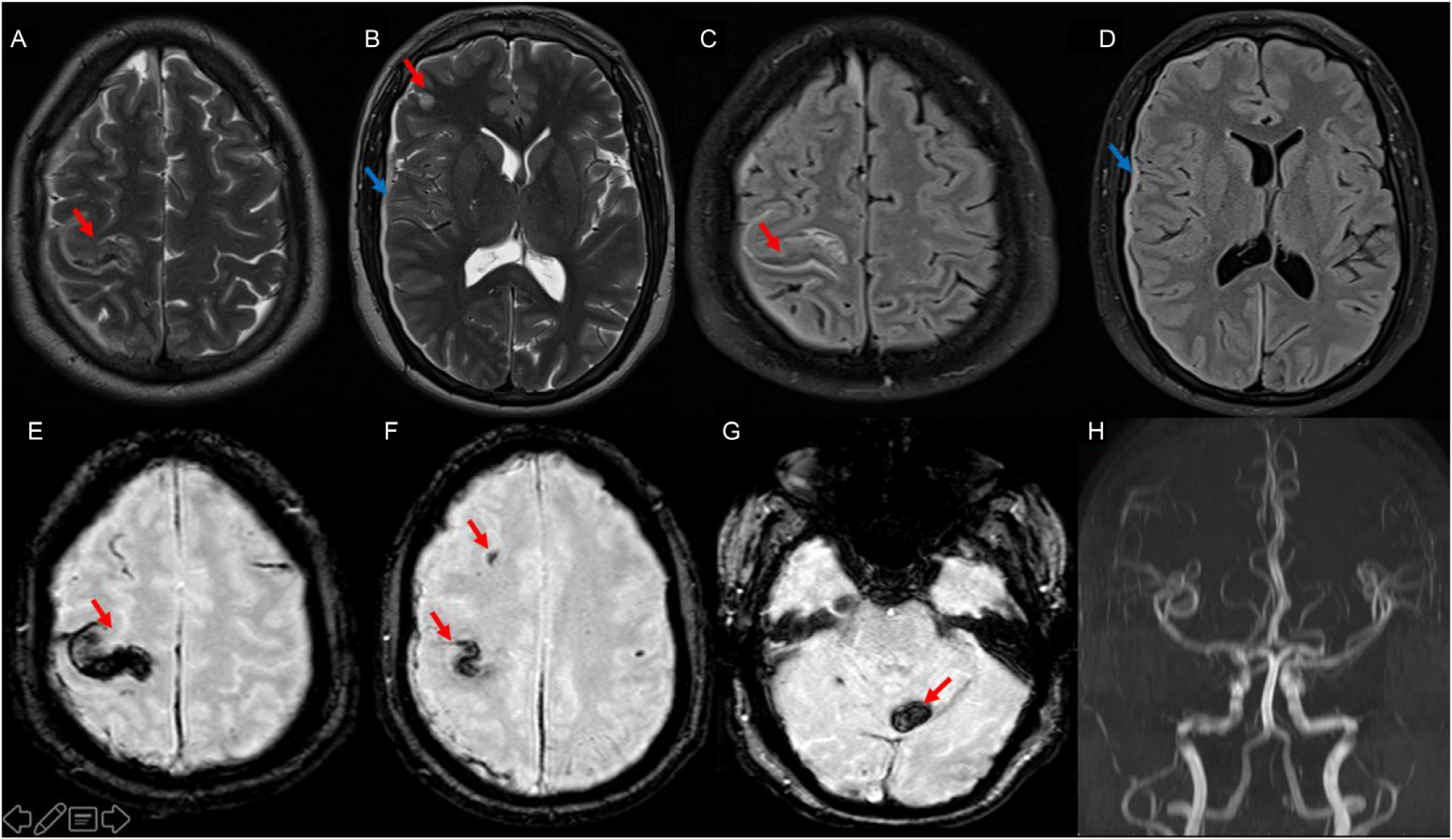
Axial MRI images: T2 (A,B), FLAIR (C,D), SWI (E–F-G), and arterial TOF (H) sequences show multiple hemorrhagic embolic lesions, hyperintense on T2/FLAIR and hypointense on SWI, with a left cerebellar hematoma (red arrows) and a right subdural hematoma (blue arrows).

No leptomeningeal enhancement was observed. The ventricular system was normal. MR angiography showed no evidence of vascular malformation or intracranial aneurysm. More than 20 punctate hypointense lesions were identified, indicating a high embolic burden. However, mycotic anevrysms may appear secondarily, justifying follow-up vascular imaging (Fig. 1).

These imaging findings were consistent with diffuse hemorrhagic septic cerebral emboli complicating infective endocarditis, associated with subarachnoid hemorrhage and a subdural hematoma.

The patient was a 40-year-old male with no known history of intravenous drug use or prior valvular disease. Blood cultures grew *Staphylococcus aureus*. Echocardiography revealed a large vegetation measuring 12 mm on the mitral valve. On admission, the Glasgow Coma Scale (GCS) score was 9/15. Neurological deterioration occurred 5 days after diagnosis of infective endocarditis. Laboratory findings showed elevated CRP and leukocytosis with normal coagulation profile. The patient was treated with targeted intravenous antibiotics. Anticoagulation was withheld due to intracranial hemorrhage. Clinical evolution showed gradual neurological improvement with GCS rising to 13/15. Follow-up MRI performed after 2 weeks demonstrated stabilization of lesions. Cardiac surgery was postponed due to hemorrhagic risk.

## Discussion

This case is particularly notable due to the simultaneous presence of subarachnoid hemorrhage and subdural hematoma, a rare association in infective endocarditis. The pathophysiology likely involves septic arteritis with vessel wall fragility, possible microaneurysm rupture, and contribution of sepsis-related mechanisms. This may also be explained by sepsis-related coagulopathy despite normal initial coagulation tests.

Septic cerebral emboli represent the most common neurological complication of infective endocarditis and are a major determinant of outcome [1,4]. They typically originate from valvular vegetations and preferentially affect cortical and subcortical territories, often in multiple vascular distributions [5].

Clinically, septic emboli may present with focal deficits, seizures, or altered consciousness. In this patient, altered consciousness was the main presentation, reflecting multifocal cerebral involvement.

MRI is highly sensitive in detecting these lesions, particularly diffusion-weighted imaging, which reveals acute ischemic embolic infarcts even in clinically silent patients [6]. In the setting of IE, embolic infarcts frequently undergo hemorrhagic transformation due to infectious arteritis, vessel wall destruction, and inflammatory-mediated fragility [3,7].

Susceptibility-weighted imaging plays a crucial role in identifying hemorrhagic components and cerebral microbleeds, which are common in IE and may be underestimated on conventional sequences [8]. Several studies have reported cerebral microbleeds in up to 50%–60% of patients with IE undergoing MRI, reflecting diffuse vascular involvement [8,9]. The extensive hemorrhagic pattern observed in our case is consistent with these findings.

Subarachnoid hemorrhage and subdural hematoma are less frequent but recognized complications. They may result from rupture of fragile septic vessels or occult infectious aneurysms, even in the absence of angiographic abnormalities at initial imaging [10]. Their presence has important therapeutic implications, particularly regarding anticoagulation and timing of cardiac surgery [11].

The absence of contrast enhancement in our case helped exclude differential diagnoses such as cerebral abscess or meningitis, while MR angiography ruled out vascular malformations. Thus, multiparametric MRI provided a comprehensive assessment of lesion type, extent, and associated complications, directly impacting patient management. However, mycotic aneurysms may appear secondarily, justifying follow-up vascular imaging.

Management relies on prolonged intravenous antibiotics adapted to microbiological findings. Anticoagulation is generally avoided in hemorrhagic forms. Surgical valve replacement may be delayed depending on neurological stability. Follow-up vascular imaging is recommended to detect delayed mycotic aneurysms [12].

## Conclusion

Diffuse hemorrhagic septic cerebral emboli are a severe neurological complication of infective endocarditis. Brain MRI, including DWI and SWI sequences, is essential for accurate diagnosis and evaluation of lesion burden. Early recognition of hemorrhagic involvement is crucial, as it significantly influences prognosis and therapeutic decisions.

## Patient consent

Written informed consent was obtained from the patient for the publication of this case report.
